# Emergence of Leptin in Infection and Immunity: Scope and Challenges in Vaccines Formulation

**DOI:** 10.3389/fcimb.2018.00147

**Published:** 2018-05-09

**Authors:** Dayakar Alti, Chandrasekaran Sambamurthy, Suresh K. Kalangi

**Affiliations:** ^1^School of Life Sciences, University of Hyderabad, Hyderabad, India; ^2^BIO5 Institute, University of Arizona, Tucson, AZ, United States

**Keywords:** leptin, obesity, malnutrition, immunity, infections, vaccination

## Abstract

Deficiency of leptin (*ob/ob*) and/or desensitization of leptin signaling (*db/db*) and elevated expression of suppressor of cytokine signaling-3 (SOCS3) reported in obesity are also reported in a variety of pathologies including hypertriglyceridemia, insulin resistance, and malnutrition as the risk factors in host defense system. Viral infections cause the elevated SOCS3 expression, which inhibits leptin signaling. It results in immunosuppression by T-regulatory cells (Tregs). The host immunity becomes incompetent to manage pathogens' attack and invasion, which results in the accelerated infections and diminished vaccine-specific antibody response. Leptin was successfully used as mucosal vaccine adjuvant against *Rhodococcus equi*. Leptin induced the antibody response to *Helicobacter pylori* vaccination in mice. An integral leptin signaling in mucosal gut epithelial cells offered resistance against *Clostridium difficile* and *Entameoba histolytica* infections. We present in this review, the intervention of leptin in lethal diseases caused by microbial infections and propose the possible scope and challenges of leptin as an adjuvant tool in the development of effective vaccines.

## Introduction

### Leptin and leptin receptors

**Figure 1:**
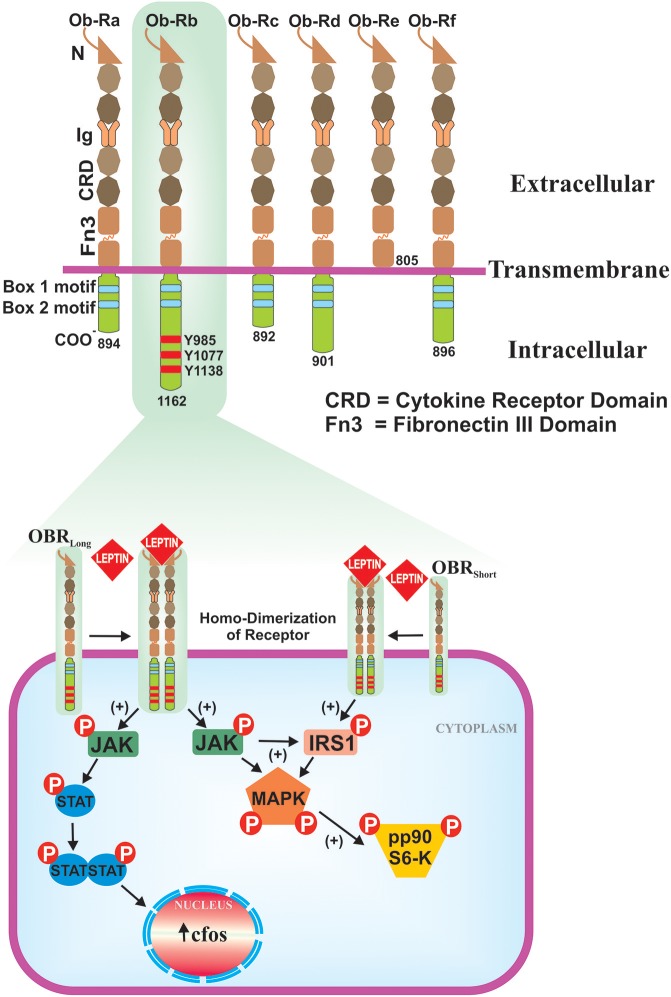
Isoforms of the leptin receptor and ob-Rb signaling pathways. Six isoforms (Ob-Ra to Ob-Rf) of LEPR are existed, all are identical in extracellular ligand binding domains but differ in C-terminus. Out of six isoforms, only Ob-Rb encodes protein motifs involve in the activation of JAK-STAT signaling pathway. Ob-Rb has three tyrosine conserved regions (Y985, Y1077 & Y1138) in cytoplasmic domain. Later, it functions as a docking site for STAT3. Binding of leptin to ob-Rb leads to receptor homodimerization, in turn activates JAK/STAT pathways that result in the activation of c-fos. Ob-Rb also phosphorylates JAK to the activation of insulin receptor substrate-1 (IRS-1) and MAPK.

Leptin was discovered as the product of obese (*ob*) gene (Zhang et al., 1994), which is located on the long arm of 7th chromosome at the position 31.3 (7q31.3) of about ~20 Kb length (Considine and Caro, 1997). The nascent non-glycosylated protein has a M.W. 16 kDa, with an NH_2_ terminal signal peptide (21-amino acids). Biologically active form of leptin is 146 amino acids peptide. Leptin freely circulates in the blood and exerts its actions via membrane bound leptin receptors (LEPRs) (Ceddia, 2005). Leptin receptor gene (*Ob*-*R*) comprises four short-isoforms (*Ob*-*Ra, Rc, Rd*, and *Rf*), one long-isoform (*Ob-Rb*) and one soluble-isoform (*Ob-Re*) (Tartaglia, 1997). Six isoforms of Ob-R are identical to each other and contain 805 amino acids and 1–14 exons present in extracellular domain. Ob-Rb isoform consist of long intracellular domain that resembles the type-I cytokine receptor signaling domain and transduces via Janus tyrosine kinase/signal transducer and activator of transcription (JAK/STAT) pathway (Houseknecht and Portocarrero, 1998; Licinio et al., 1998). In fact, most of the biological functions of leptin are exerted by Ob-Rb-JAK/STAT signaling cascade, which is present predominantly in hypothalamus but has moderate presence in other tissues. Ob-Ra and Ob-Rc isoforms regulate the transportation of leptin across the blood-brain barrier (BBB) to hypothalamus and Ob-Rf also performs the same function to a lesser extent. Ob-Re isoform is a soluble binding protein and lacks the transmembrane and cytoplasmic domains (Figure 1). It binds to the plasma leptin and inhibits its glomerular clearance, but it does not interfere with the binding of leptin to Ob-Rb. Ob-Re infusion into *ob/ob* null mice enhances the activity of leptin and its overexpression independent of the adipose leptin expression (Huang et al., 2001).

Functionally, leptin is a hormone derived from adipocytes (La Cava and Matarese, 2004), in response to the food intake and energy balance. It conveys the signals to the hypothalamus/central nervous system (CNS) and peripheral organs, to maintain the metabolic homeostasis. The systemic leptin concentrations are influenced by the total fat mass (Grinspoon et al., 1996) and body mass index (BMI). They are also influenced by metabolic hormones, sex, and body energy demands. Congenital leptin deficiency in humans is rare and limited to <5%, reported in obese population in the United States. Diet-induced obesity in humans' results in the increase in systemic leptin levels and its resistance is due to the desensitized LEPRs (Burguera et al., 2000; Morrison, 2008); conversely, the systemic leptin levels are reduced in malnutrition and in starvation. Malnutrition is a burning issue and affects around 826 million people of the world population (Katona and Katona-Apte, 2008). Children, <5 years of age, are the major victims of malnutrition, which accounts for 2.2 million annual deaths globally (Black et al., 2008). Malnutrition affects both innate and acquired immunity (Woodward, 1998; Schaible and Stefan, 2007), and the ratio of CD4+/CD8+ T cells (Chandra, 1992). People with malnutrition are vulnerable to infections because of immunosuppression (Faggioni et al., 2000b) and defective cytokine production (Zhang et al., 1997).

In this article, we discuss in detail about the leptin dependent regulation of immune responses, relation between microbial infections and leptin signaling; and discuss the potential of leptin as vaccine adjuvant.

## Leptin in immune system

Leptin bridges a link between nutrtional status and immune system of individuals. Human leptin has four α-helices similar to that of long-chain α-helical cytokine family, which includes interleukin (IL)-6, IL-11, IL-12, leukemia inhibitory factor (LIF), granulocyte-colony stimulating factor (G-CSF), ciliary neurotrophic factor (CNTF), and oncostatin (Zhang et al., 1997). LEPRs exhibit homology with the glycoprotein (gp)-130 signal transducing subunit of the IL-6 type cytokine receptor (Tartaglia et al., 1995; Baumann et al., 1996; Lee et al., 1996). Because of the structural similarities with the above-mentioned immune components, leptin acts as a cytokine. It is also called “adipokine” since it is derived from adipose tissue. As an adipokine, leptin regulates the normal development of hematopoiesis, angiogenesis, and innate & adaptive immunity (Loffreda et al., 1998; Santos-Alvarez et al., 1999; Martín-Romero et al., 2000; La Cava and Matarese, 2004; Matarese et al., 2005) (see Table 1). In immune cells, leptin and LEPRs predominantly activates JAK2/STAT3 signaling cascade, in which the phosphorylated tyrosine-1138 (pTyr^1138^) of Ob-Rb intracellular domain acts as a docking site for STAT3 (Papathanassoglou et al., 2006). Beside this, leptin also triggers SH2-containing tyrosine phosphatase (SHP2)-dependent mitogen-activated protein kinase (MAPK) and phosphoinositide 3-kinase/serine/threonine protein kinase/mammalian target of rapamycin (PI3K/Akt/mTOR) pathway in which, the pTyr^985^ residue acts as a docking site for SHP-2 (Fernández-Riejos et al., 2010). Leptin signaling is inhibited by the overexpression of SOCS3 (Krebs and Hilton, 2001), which affects JAK/STAT pathway by binding to the pTyr^985^ of Ob-Rb and induces dephosphorylation of JAK2 (Bjørbæk et al., 2000). Protein tyrosine phosphatases (PTPs), the phosphatase and tensin homolog (PTEN), receptor-type PTPe (RPTPe), and PTP1B also induce dephosphorylation of JAK2 and inhibit leptin signaling. The expression of PTP1B and T cell PTP (TCPTP) upregulates in high-fat diet and obesity, and inhibits leptin-mediated STAT3 phosphorylation (St-Pierre and Tremblay, 2012). The PTP1B mediated endoplasmic reticulum (ER) stress induces leptin resistance (Hosoi et al., 2008; Ozcan et al., 2009), possibly by inhibiting STAT3 phosphorylation.

**Table 1:** Adipokine action of leptin on the cells of both innate and adaptive immunity.

**Immune cell type**	**Observation**	**References**
**LEPTIN ACTION ON DIFFERENT CELLS OF INNATE IMMUNITY**		
Neutrophils	+ Activation	La Cava and Matarese, 2004
	+ Chemotaxis	Mancuso et al., 2002
	+ Phagocytosis	Fernández-Riejos et al., 2010
	+ H_2_O_2_, ${\text{O}}_{2}^{-}$ production	Caldefie-Chezet et al., 2001
Monocytes/Macrophages	+ Proinflammatory cytokines (IL-1, IL-6, TNF) production +Phagocytosis + Leukotrienes B4 + Antigen presentation + MHC expression + Acute inflammation + Adhesion molecules	Loffreda et al., 1998 Santos-Alvarez et al., 1999 Bruno et al., 2005 La Cava and Matarese, 2004
Natural Killer cells	+ Cytotoxicity + Perforin production + IL-2 secretion	La Cava and Matarese, 2004 Tian et al., 2002
Dendritic cells	+ Activation + IL-12 secretion + Th1-priming	Spencer and Daynes, 1997 Mattioli et al., 2005
**LEPTIN ACTION ON DIFFERENT CELLS OF ADAPTIVE IMMUNITY**		
Thymic T-cells	+ Proliferation + Maturation & Homeostasis + CD4+CD8+ & CD4+CD8- + Bcl-2 & Bcl-XL, − Apoptosis	Matarese et al., 2005 Howard et al., 1999 Bruno et al., 2005
Naive T-cells (CD4+CD45RA+)	+ Proliferation + Activation of MAPK & PI3K + IL-2 production	Lord et al., 1998
Memory T-cells (CD4+CD45RO+)	– Proliferation	Lord et al., 1998
Th1-cells	+ Stimulation + IFN-γ & TNF production + DTH response + IgG2a switching by B-cells	Martín-Romero et al., 2000 Napoleone et al., 2007
Th2-cells or Tregs	+ Inhibition + IL-4 & IL-10 – IgG1 switching by B-cells	Martín-Romero et al., 2000 Napoleone et al., 2007
Cytotoxic T-cells	+ Activation & proliferation + Granzyme production – PD-1, CTLA-4 expression	Dayakar et al., 2017

In innate immunity, leptin modulates the activity and function of neutrophils by increasing chemotaxis and secretion of oxygen radicals such as hydrogen peroxide (H_2_O_2_) and superoxide (${\text{O}}_{2}^{-}$). In mice, directly acts on neutrophils, whereas, in humans, its action is mediated by tumor-necrosis factor (TNF) secreted from monocytes. The stimulation of human monocytes by leptin induces the production of TNF-α and IL-6 (Santos-Alvarez et al., 1999). Leptin enhances phagocytosis by macrophages, secretion of pro-inflammatory mediators of acute-phase response, and expression of adhesion molecules. In natural killer (NK) cells, it increases cytotoxic ability and secretion of perforin and IL-2. In adaptive immunity, leptin promotes the generation, maturation, and survival of thymic T cells, Leptin inhibits the apoptosis of thymocytes by inducing tyrosine phosphorylation of sam68 and insulin receptor substrate (IRS)-1, which associates with p85 regulatory subunit of SH2-domain, and PI3K signaling (Sung et al., 1994; Sánchez-Margalet and Najib, 1999). In naive T cells, leptin increases proliferation and IL-2 secretion via the activation of MAPK and PI3K pathways. In memory T cells, leptin triggers Th1 polarization by increasing interferon (IFN)-γ and TNF-α secretion. It also promotes delayed type hypersensitivity (DTH) response and immunoglobulin class-switching to produce IgG2a. This process sustained by an autocrine loop of leptin secretion by Th1 cells. Leptin has anti-apoptotic effects on mature T cells and hematopoietic precursors (La Cava and Matarese, 2004).

Studies on *ob/ob* mice revealed that they produce low amount of IL-2, IFN-γ, and IL-18, and large amount of Th2 cytokines (IL-4 and IL-10). Leptin regulates thymic homeostasis and induces Th1 response by increasing the production of IFN-γ and TNF-α, which in turn activates monocyte or macrophages and dendritic cells (DCs) (Zhang et al., 1997; Loffreda et al., 1998; Santos-Alvarez et al., 1999; Martín-Romero et al., 2000; Matarese et al., 2005; Mattioli et al., 2005). Leptin induces granulocyte-macrophage colony stimulating factor (GM-CSF) and G-CSF production from murine peritonial macrophages (Gainsford et al., 1996) and leptin-deficient mice exhibit phenotypic abnormalities in macrophages (Lee et al., 1999). Leptin signaling in immature DCs upregulates surface expression of chemokine receptor CCR7 and induces structural changes by facilitating the remodeling of actin microfilaments in the cytoskeleton and in turn migratory potential of DCs (Mattioli et al., 2008). Leptin directly activates DCs to secrete IL-12, which is a key cytokine that facilitates the shifting of T cells toward Th1 phenotype (Spencer and Daynes, 1997). The *ob/ob* mice have shown impaired function of DCs and their altered steady-state number (Macia et al., 2006) and *db/db* mice have shown obese diabetic phenotype with increased number and suppressive function of Tregs (Taleb et al., 2007). Leptin stimulates the expression of toll-like receptor (TLR)-2 on primary human monocytes in hyperleptinemia to potentiate innate immunity and inflammation but had no effect on TLR-4. Leptin induces CD14 expression on THP-1 monocytes but not on primary human monocytes to potentiate lipopolysaccharide (LPS) mediated proinflammatory cytokine response (Jaedicke et al., 2013). An impaired cytotoxic activity of NK cells, lymphopenia, increased number of blood monocytes, reduced antigen-specific T cell proliferation, and thymic atrophy are common features to both *ob/ob* and *db/db* mice (Fernández-Riejos et al., 2010). These findings are consistent in human patients with congenital leptin-deficiency (Farooqi et al., 2007). On the other hand, malnutrition induces anti-inflammatory cytokines IL-4 and IL-10 and impairs pro-inflammatory cytokines IL-2 and IFN-γ production from CD4+ and CD8+ T cells in children. However, leptin administration in these children substantially reversed the above effects and induces the expression of CD25 and CD69 activation markers on peripheral blood T cells (Rodríguez et al., 2007). A drastic fall in systemic leptin levels during starvation for 48 h increases susceptibility to LPS and TNF-induced toxicity in mice. However, leptin replacement therapy markedly reverses these deleterious effects and protects the mice from fasting-induced lymphopenia (Faggioni et al., 2000b). Both malnutrition and fasting are associated with nutrients insufficiency, which limits the availability and uptake of glucose by effector T cells to fuel their maintenance and activity results in immunosuppression and incompetency to produce inflammatory cytokines. In fasting animals, an intraperitoneal injection of leptin induces the activated T cell's glucose-uptake by upregulating the expression of glucose transporter-1 (Glut1) and intrinsic glucose metabolism through glycolysis and mitochondrial respiration, which results in activated T cell proliferation and inflammatory cytokines production. However, such a metabolic regulation in naive T cells and Tregs did not require the leptin action. On the other hand, at sufficient nutritional level, an abundant expression of leptin receptors and peripheral T cell activation facilitates leptin signal to metabolically license T cells for activation. In such a way that, leptin links the nutritional status with immunity by regulating the intrinsic metabolism of T cells (Saucillo et al., 2014).

Leptin induces the expression of CD25 and human leukocyte antigen (HLA)-DR on peripheral blood B-lymphocytes and in turn activates JAK2/STAT3and p38MAPK/ERK 1/2 to produce IL-6, IL-10, and TNF-α (Agrawal et al., 2011). Leptin receptor expression was significantly higher in Tregs compared to CD4+CD25- effector T cells and leptin directly acts on Tregs to inhibit their function and proliferation (De Rosa et al., 2007). The persistence of high systemic leptin levels triggers the mTOR signaling to inhibit Tregs proliferation. Tregs-derived leptin acts in autocrine fashion to maintain their hyporesponsiveness. In *db/db* mice, Tregs show a decreased mTOR activity and increased proliferation (Procaccini et al., 2012). Leptin regulates the survival and proliferation of autoreactive CD4+ T cells by modulating the activity of Bcl-2 and Th1/Th17 cytokines via nutrient/energy-sensing Akt-mTOR pathway (Galgani et al., 2010). In animal models with autoimmunity and infectious diseases, leptin regulates Th1/Th2/Treg balance to control the disease (Procaccini et al., 2012). Acute leptin during infection and inflammation may be a protective module of the host response to inflammation (Sarraf et al., 1997). Leptin induces the proliferation of naive T cells (CD4+CD45RA+) but inhibits memory T cells (CD4+CD45RO+) in mice model (Lord et al., 1998). Leptin facilitates a survival signal to CD4+CD8+ and CD4+CD8– T cells during maturation (Howard et al., 1999). T cells skewing toward Th1 response by leptin seems to be mediated by inducing the synthesis of IL-2, IL-12, and IFN-γ and inhibiting the production of IL-10 and IL-4 (Martín-Romero et al., 2000; Napoleone et al., 2007). The isoform type and quantity of LEPRs expression on immune cells is based on cell type. For example, Ob-Rb expresses on 25% of monocytes and 12% of neutrophils (Zarkesh-Esfahani et al., 2001). A variety of functions of immune cells mediated by leptin is orchestrated by different isoforms of LEPR and CD4+ T cell population alone express three different isoforms of LEPR (Walduck and Becher, 2012). Therefore, leptin shows differential activity on CD4+ effector T cells and Treg cells at the same time.

## Leptin induces phagocytosis in microbial infections

Phagocytosis is a key event executed by certain immune cells to internalize the foreign particles (may be pathogen) inside the cell and subsequent killing. Leptin induces phagocytic activity of macrophages and prevents the apoptosis of a variety of immune cells involved in innate and adaptive immune response by delaying the cleavage of *Bid* and *Bax*, release of cytochrome-c from mitochondria, and activation of both caspase-8 and caspase-3 (Bruno et al., 2005). Upon binding to the Ob-Rb expressed on the cell surface, leptin can induce the phagocytic activity of neutrophils (Fernández-Riejos et al., 2010). Leptin was able to induce the macrophages phagocytic activity caused by *Leishmania major* infection in mouse model (Gainsford et al., 1996) and *in vitro L. donovani* infection in human macrophages (THP-1) and peripheral blood mononuclear cells (PBMCs) (Dayakar et al., 2016). Leptin stimulates neutrophils to increase the intracellular reactive oxygen species (ROS) within the phagosomes (Caldefie-Chezet et al., 2001; Fernández-Riejos et al., 2010). It activates the phagosomes by inducing actin polymerization and rejuvenating actinomyosine interaction (Attoub et al., 2000), which eventually enhances neutrophilic phagocytosis (Mancuso et al., 2002; Shirshev and Orlova, 2005). During *Klebsiella pneumonia* infection, leptin knock-out mice exhibited defective phagocytic response, in which, neutrophils have shown decreased expression of CD11b on their surface. With exogenous leptin supply, the CD11b expression was normalized, and CD11b-dependent phagocytosis induction by leptin in neutrophils enabled the animals to recover from *K. pneumonia* infection (Moore et al., 2003). Leptin induced CD11b expression and phagocytosis in neutrophils during uptake of *Listeria monocytogenes* was mediated by TNF-α produced from monocytes (Tian et al., 2002). In *Escherichia coli* infection, leptin-deficiency impaired the phagocytosis by peritoneal macrophages; *a*dequate supply of leptin inhibited the normal lymphocytes apoptosis by FAS-mediated pathway, and protected the starved mice from the loss of lymphocytes (Merrick et al., 1997). Steroids induced apoptosis in lymphocytes was reversed by leptin administration (Papathanassoglou et al., 2006). *K. pneumonia* infection has amplified the mortality rate in leptin-deficient mice by impairing bacterial clearance, and an *in vitro* study demonstrated that leptin supplementation reduced this infection by increasing the phagocytic activity of alveolar macrophages. Pre-treatment of peritoneal macrophages of leptin-deficient mice with higher concentrations of murine leptin restored the cysteinyl-leukotriene synthesis, which helped in improving phagocytosis. The systemic administration of leptin by intraperitoneal route in septic *ob/ob* and septic wild-type mice improved the neutrophil phagocytosis of *E. coli* by 21 and 10% respectively compared to untreated *ob/ob* mice (Tschöp et al., 2010).

Leptin induces the phosphorylation of Akt kinase and intracellular ROS production from *L. donovani* infected THP-1 to stimulate the macrophage phagocytic activity (Dayakar et al., 2016). In consistence, similar results were published with sodium-antimony gluconate treatment to *L. donovani* infection, which is a first-line drug to cure visceral leishmaniasis (VL) in the Indian subcontinent (Basu et al., 2006). As mentioned-above in neutrophilic phagocytosis, the intracellular ROS helps in rapid internalization of parasites by the host cells via inducing the fusion of phagosome with lysosome (Gueirard et al., 2008) for oxidative killing (Laufs et al., 2002). Leptin was also shown to promote the host defense in pulmonary bacterial infection of mouse model (Mancuso et al., 2002). These studies support the view that leptin administration could be a novel approach for protection against the bacterial infections, and may prove beneficial to human population susceptible to bacterial pneumonia under certain pathological conditions like HIV infection, malnutrition, and diabetes mellitus (Jubiz et al., 1984; Skerrett et al., 1990; Coffey et al., 1996; Cederholm et al., 2000).

## Fate of leptin in microbial infections

Generally, leptin-deficiency is associated with enhanced susceptibility to several infections. At the same time, certain infections also caused the downregulation of systemic leptin levels and mimics malnutrition like situation. Fever, diarrhea, malabsorption, low appetite, loss of nitrogen, and nutrients induce the malnutrition state and increase the mortality rate by infections (Müller et al., 2003). A few such infections; bacteria (Table 2A), virus and fungus (Table 2B), and parasite (Table 2C) infections and their pathogenicity in either low systemic leptin or leptin-deficiency and impaired leptin signaling conditions are presented below in detail.

**Table 2:** Leptin and/or its signaling associated events in multiple microbial infections.

**(A) Leptin vs. bacterial infections**		
**Infection type**	**Observation**	**References**
***Mycobacterium tuberculosis*** **(TB)**	Low systemic leptin levels During leptin deficiency; Defective granulomas Reduced CD4+ & CD8+ T-cell proliferation & activation Impaired DTH response Depleted IFN-γ levels Increased disease severity	van Crevel et al., 2002 Wieland et al., 2005
***Klebsiella pneumonia*** **(Pneumococcal pneumonia)**	During leptin-deficiency; High susceptibility Impaired leukotriene synthesis & phagocytosis by neutrophils Low CD11b on neutrophils Impaired bacterial clearance & increased mortality	Moore et al., 2003 Mancuso et al., 2002
***Streptococcus pneumoniae*** **(Pulmonary pneumonia)**	During leptin-deficiency; Impaired phagocytosis by alveolar macrophages Impaired killing by PMNs High TNF-α, MIP-2, PGE_2_ in the lung Failure of host defense system	Hsu et al., 2007
***Clostridium difficile***	Q223R	
**(Colitis)**	(rs1137101)mutation;	
	Impaired Stat3 signaling Inadequate inflammation High rate of infection ob-Rb intracellular domain Tyr 1138 Ser mutation; Switch on Stat3/SOCS3 signaling low chemokines production & immune cells recruitment	Madan et al., 2014
**Sepsis (SIRS)**	Leptin deficiency; Highly fatal CNS leptin signaling induces protective immunity against this infection	Takahashi et al., 2004 Tschöp et al., 2010
***Staphylococcus aureus*** **(Sepsis arthritis)**	Reduced leptin production Higher susceptibility & IL-6 levels in *db/db*	Hultgren and Tarkowski, 2001
***Helicobacter pylori*** **(Gastric ulcers)**	Increased gastric leptin No change in systemic leptin Reduced gastric leptin after cure *Db/db* exhibited high susceptibility	Azuma et al., 2001 Khosravi et al., 2015
**Bacterial peritonitis** ***Listeria monocytogenes***	LEPR Q223R mutation in CRH1 domain higher susceptibility to infection Downregulation of MCP-1 in *db/db* and *ob/ob*, and KC mRNA n *db/db* mice liver	Bracho-Riquelme et al., 2011 Ikejima et al., 2005
**(B) Leptin vs. virus and fungal infections**		
**Infection type**	**Observation**	**References**
**HIV (AIDS)**	Increased expression of LEPRs on monocytes Low systemic leptin levels Leptin inhibited ROS and oxidative burst by HIV+ monocyte Monocytes desensitization & Impaired immunity During anti-retroviral therapy leptin positively correlated with CD4+ T-cells Induced SOCS3 expression Inhibited IFN-α/β JAK/STAT signaling	Sánchez-Margalet et al., 2002 Estrada et al., 2002 Sánchez-Pozo et al., 2003 Najib and Sánchez-Margalet, 2002 Karp et al., 1998 Azzoni et al., 2010 Matarese et al., 2002 Akhtar et al., 2010
**Influenza A/H1N1 Pneumonia**	Acute raise in pulmonary leptin levels Increased neutrophil survival Increased neutrophilia In obesity, global reduction of LEPRs Reduced viral clearance Impaired CD8+ T-cell memory Induced SOCS3 expression	Ubags et al., 2014 Morgan et al., 2010 Radigan et al., 2014 Karlsson et al., 2010 Pauli et al., 2008
**Adenovirus**	Induced obesity Increased risk of influenza Decreased leptin & nor-epinephrine Increased appetite & glucose uptake Decreased lipolysis	Hur et al., 2013
**Hepatitis-B & Epstein Barr virus**	During obesity / hyperleptinemia; Induced SOCS3 expression Inhibited IFN-α/β JAK/STAT signaling	Michaud et al., 2010 Tian et al., 2012
**Encephalomyocarditis**	Impaired expression of cardiac adiponectin Induced expression of TNF-α Severe inflammatory myocardial damage	Takahashi et al., 2006
**Coxsackie virus B4**	Higher susceptibility in *db/db* animals	Webb et al., 1976
***Candida albicans*** **(Fungus)**	Leptin receptor-deficiency in obesity;	
	Impaired immunity During stress; low systemic leptin levels High TNF-α levels	Rodríguez-Galán et al., 2010
**(C) Leptin vs. intra and extracellular parasite infections**		
**Infection type**	**Observation**	**References**
***Entameoba histolytica*** **(Amoebiasis)**	Low serum leptin levels in amoebic liver abscess LEPR Q223R mutation in CRH1 domain; High disease severity LEPR E233R mutation; High susceptibility 2012 Leptin deficiency; Intermediate susceptibility Intensive epithelial denudation Integral leptin signaling protects via Sat3 and Erk or Akt pathways Leptin promotes regeneration & mucin secretion from intestinal epithelium Inhibits apoptosis & maintains integrity in intestinal epithelium	Alam et al., 2016 Duggal et al., 2011 Vedantam and Viswanathan, 2012 Sukhotnik et al., 2009 El Homsi et al., 2007 Brun et al., 2007
***Leishmania major*** **&** ***L. donovani***	Low serum leptin levels Reduced phagocytosis by macrophage	
	Leptin induces pErk1/2 & pAkt in macrophages & Phagocytosis Increased Th1 cytokine response Induced protective immunity in *ob/ob* liver Increased IFN_γ_, IL12, IL1β Increased CD8+ T-cell count Increased IgG2a levels Decreased IgG1 levels Increased granuloma Repaired tissue degeneration Controlled weight loss Reduced parasite load Reduced PD-1 & CTLA-4 expression	Dayakar et al., 2016 Gainsford et al., 1996 Shivahare et al., 2015 Maurya et al., 2016 Dayakar et al., 2017
***E. Histolytica*** **&** ***Giardia***	Higher leptin levels Damage of gut epithelial cells	
	Activation of mesenteric lymphnodes & adipose tissue Eosinophilia & extensive tissue invasion & High pathogenicity	Desreumaux et al., 1999 Yahya et al., 2016
***Taenia taeniaformis***	Low systemic leptin levels	Krebs and Kacelnik, 1991
	Anorexia High PGE2 & Inhibition IL-12 & Th2 immunity Inhibition of skin Langerhans cells to lymphnodes	Lõhmus and Sundström, 2004 Leid and McConnell, 1983
***Heligmosomoides bakeri***	Higher serum leptin levels Acute inflammation High IL-1β, TNF-α, and IL-6	Tu et al., 2007 Noah et al., 1994
**Malaria**	Higher serum leptin levels	Pulido-Mendez et al., 2002

### Plasma leptin levels and disease severity of pulmonary infections

Several studies reported that plasma leptin levels are lower in TB patients than in healthy controls. Certain clinical conditions like muscle wasting, inflammation, and decreased energy intake in TB patients results in high severity of disease (van Crevel et al., 2002). Furthermore, leptin levels were higher in female patients with higher body fat mass than in male patients with equal body weights suffering with TB infections (Çakir et al., 1999). Even at the normal physiological conditions, leptin levels (5–10 ng/ml) were two-folds higher in females compared to those of males at equal BMI (Thomas et al., 2000). Leptin-deficient mice with pulmonary TB were deficient in CD4+ and CD8+ T-lymphocytes number and activation. They were unable to generate IFN-γ and DTH responses, the exogenous leptin supply restored the lymphocyte trafficking, IFN-γ production, and granuloma formation successfully, and disease severity was substantially reduced (Wieland et al., 2005). Leptin-deficient mice also exhibited greater susceptibility and prolific lethality to pneumonia infection, caused by either *K. pneumonia* or *Streptococcus pneumoniae*. Leptin supply to *ob/ob* animals improved host defense against pneumococcal pneumonia caused by *K. pneumonia* by increased pulmonary bacterial clearance and host survival. Pulmonary bacterial load during *S. pneumonia* infection in leptin-deficient individuals results in the failure of host defense system, which is associated with abundant production of TNF-α, macrophage inflammatory peptide (MIP)-2 and prostaglandin-E_2_ (PGE_2_) in the lung tissue. The key innate immunity events like phagocytosis by alveolar macrophages and killing by neutrophil polymorphonuclear leukocytes (PMN) were impaired. It results in successive evasion of *S. pneumonia* infection, *in vitro*. However, exogenous leptin has tremendously improved the survival rates in leptin-deficient mice- by inducing substantial clearance of bacteria from pulmonary tissue as well as from blood (bacteraemia). Likewise, *in vitro S. pneumonia* infection was controlled by exogenous leptin through inducing reconstitution of alveolar macrophage phagocytosis and PMN-mediated H_2_O_2_ production (Hsu et al., 2007).

### Role of leptin in mucosal immunity

*C. difficile* is a leading cause of nosocomial infection, which results in the development of colitis. Earlier studies have reported that Q-to-R mutation at position 223 in the LEPR cytokine receptor homology 1 (CRH1) domain (LEPR Q223R) is associated with increased susceptibility to bacterial peritonitis (Bracho-Riquelme et al., 2011) and to infectious colitis as well as liver abscess caused by *E. histolytica* in humans (Duggal et al., 2011). Similarly, the risk of *C. difficile* infection in humans is higher in LEPR Q223R (rs1137101) model, which is a homozygous allelic mutation that results in impaired STAT3 signaling and increased dissemination of infection. In murine model, the mechanism of susceptibility to *C. difficile* was elucidated as the animals being deficit in functional LEPR and adequate inflammation to clear the infection from the gut lumen. In addition to this, a mutation (Tyr 1138 Ser) in tyrosine 1138 residue located in the intracellular domain of LEP-Rb isoform mediates STAT3/SOCS3 signaling, which results in decreased chemokine production and immune cells recruitment at the site of infection in mucosal gut tissue. Leptin was shown to be protective against *C. difficile* colitis by inducing STAT3 inflammatory pathway, which is impaired in LEPR Q223R mutation (Madan et al., 2014). Similarly, the *db/db* mice exhibited greater susceptibility to *H. pylori* infection. In humans, *H. pylori* infection substantially increased the localized gastric leptin levels but their levels were significantly diminished after cure. However, the systemic circulating leptin levels in serum were not altered during this infection (Azuma et al., 2001). Similarly, in pigs, the enteric *Salmonella typhimurium* challenge did not alter the serum leptin levels (Jenkins et al., 2004). In another study, *H. pylori* infection induced the gut leptin levels in specific pathogen-free mice by interacting with gut microbiome (Khosravi et al., 2015).

### Leptin offers protection against sepsis

Sepsis is another systemic inflammatory response syndrome (SIRS) in certain infection states (Santos-Alvarez et al., 1999). It is responsible for multiple organ failure and high rate of mortality. Leptin deficiency is highly fatal in mice suffering from sepsis due to severe organ damage (Takahashi et al., 2004). Systemic leptin replacement modulated the immune response against sepsis and increased survival in both leptin-deficient and wild-type mice. It has also been reported that endogenous CNS leptin signaling is necessary to induce adequate anti-septic immune response. In sepsis, the leptin infusion via intracerebroventricular route into the CNS of *ob/ob* mice significantly reduced IL-6 levels in serum thereby controlled systemic inflammation and improved survival. Genetic rescue of leptin signaling in the CNS of *db/db* mice improved the survival in sepsis compared to non-rescued *db/db* mice. In humans, three-fold higher leptin levels were reported in the patients' recovered from sepsis compared to that of non-survivors. These observations reveal the neuroendocrine regulation of systemic immunity and therapeutic potential of leptin in infectious disease (Tschöp et al., 2010). Septic arthritis is caused by *Staphylococcus aureus*; leptin production was found to be downregulated during this infection, in murine model. The *db/db* mice exhibited greater susceptibility to *S. aureus* infection. Though exogenous recombinant leptin supply failed in restoring the basal leptin levels and clearance of bacterial load in these animals, it substantially reduced the severity of septic arthritis by regulating the production of inflammatory cytokine IL-6 (Hultgren and Tarkowski, 2001).

### Leptin offers resistance to *L. monocytogenes* infection

*L. monocytogenes* is an intracellular bacterium, which severely affects immunodeficient individuals and neonates to causing listeriosis, meningitis, and endocarditis (Economou et al., 2000; Portnoy et al., 2002; Chan et al., 2005). “Listeriolysin O” produced by this bacterium inhibits antigen-processing by inducing the lysis of phagosome membrane in macrophages, which results in abundant intracellular growth of bacteria (Carrero et al., 2004; Birmingham et al., 2008). In addition to this, “Listeriolysin O” also cause death in antigen-presenting cells (APCs) by inducing the production of inflammatory mediators (Savill et al., 1993; Carrero and Unanue, 2006) and this bacterial infection leads to the apoptosis in T-lymphocytes (Leib et al., 1996; Zychlinsky and Sansonetti, 1997). The *db/db* and *ob/ob* mice were highly susceptible to this infection and incapable to clear the bacteria from liver. The hepatic infection of *L. monocytogenes* downregulates the expression of chemokines such as monocyte chemoattractant protein (MCP)-1 and KC mRNA in *db/db* mice and MCP-1 in *ob/ob* mice when compared to their heterozygote (*db/m* and *ob/?*) phenotypes. Leptin administration in *ob/ob* mice restored the expression of MCP-1 and offered the resistance to *L. monocytogenes*. On the other hand, insulin treatment in *db/db* mice restored the expression of MCP-1 and KC mRNA and enhanced the rate of infection clearance. The hyperglycaemia in leptin-deficiency impairs the host system to clear the bacteria from liver but leptin therapy may corrected the blood glucose levels by increasing insulin sensitivity and improved host resistance to this bacterial infection (Ikejima et al., 2005).

### Leptin diminishes oxidative burst in HIV+ monocytes

Generally, certain levels of leptin can stimulate the monocytes to produce ROS via activation of membrane-bound NADPH oxidase (Rossi, 1986). Intracellular ROS can act as a second messenger in LEPR signaling of monocytes (Martín-Romero and Sánchez-Margalet, 2001; Sanchez-Margalet and Martin-Romero, 2001) and also maintain acute proinflammatory conditions (Chaudhri and Clark, 1989). It was reported that the HIV infection can also induce ROS production from monocytes and macrophages (Kimura et al., 1993; Trial et al., 1995; Elbim et al., 1999). The ROS production is an indicator of programmed cell-death in monocytes (Um et al., 1996). Interestingly, HIV+ monocytes have increased expression of LEPRs, displaying their hyperactive state (Matarese et al., 2002). However, leptin stimulation of these HIV+ monocytes partially inhibited the production of ROS (Sánchez-Pozo et al., 2003), this could be either the desensitization of HIV+ monocytes for leptin as observed in other hyper-inflammatory states such as sepsis, in which the monocytes skewed into hypo-inflammatory/anergy state by LPS stimulation (Karp et al., 1998) and results in the attenuation of oxidative burst (Von Knethen and Brüne, 2001) or the consistency of anti-apoptotic function of leptin with the inhibition of oxidative burst in HIV+ monocytes (Najib and Sánchez-Margalet, 2002). The low systemic leptin levels in HIV patients (Estrada et al., 2002) due to reduced adiposity may contribute to immunodeficiency (Kotler et al., 1985). However, during active anti-retroviral therapy, leptin levels were correlated with CD4+ T cells in HIV patients (Matarese et al., 2002).

### Leptin resistance or impaired signaling induces SOCS3 and susceptibility to virus infections

It was demonstrated that the impaired leptin signaling is attributed to defective immunity for influenza A/H1N1 (Morgan et al., 2010) and HIV infections (Azzoni et al., 2010) due to the loss of interdisciplinary regulation among immunologic, metabolic, and neuro-endocrinological aspects. However, an acute induction of pulmonary leptin levels during H1N1 pneumonia in both mice and human models directly increased the neutrophilia and neutrophils survival without inducing any secondary cytokines (Ubags et al., 2014). Global reduction of the LEPRs, results in reduced viral clearance and worse outcomes against influenza A challenge in obese mice. However, these outcomes are not specific to the reduced LEPR in lung epithelial cells or macrophages but may be associated with impaired leptin signaling in non-myeloid populations such as NK and T cells (Radigan et al., 2014). For instance, during influenza virus infection in diet-induced obesity, the CD8+ T cell memory was depleted and pulmonary SOCS3 mRNA expression was induced when compared to infected lean mice (Karlsson et al., 2010). Apart from influenza, other viruses such as hepatitis-B, HIV, and Epstein Barr virus can also induce SOCS3 expression to ensure their survival and evade the host immunity by inhibiting IFN-α/β JAK/STAT signaling (Pauli et al., 2008; Akhtar et al., 2010; Michaud et al., 2010; Tian et al., 2012). IFNs are potent anti-viral substances, capable of inducing maturation and activation of DCs, and bridging both innate and adaptive immunity, is in contrast to the function of SOCS3, which typically inhibits T cells proliferation and activation by directly targeting CD28 (Kubo et al., 2003). Altogether, the induced SOCS3 levels either in viral infection or hyperleptinemia state of over-nutrition directing to host immune dysfunction. Therefore, in obesity the potential failure of vaccination, especially against viral infections could be regulated by SOCS3 antagonists. A recent study proposed that the saponins derived from fungal endophytes could be potential inhibitors of leptin and may repair its resistance in obesity, and may modulates immune response in favor of host against multiple diseases (Mouli et al., 2016). On the other hand, adenovirus infection can induce obesity, which is a high risk factor for influenza caused morbidity and mortality. During adenovirus infection induced obesity, the cellular uptake of glucose is induced, and simultaneously lipolysis is reduced through the stimulation of corticosterone secretion. In addition, this infection may increase the appetite and substantially decrease the levels of nor-epinephrine and leptin, which tends to immune dysfunction (Hur et al., 2013).

During leptin-deficiency, encephalomyocarditis virus infection in mice contributed to the development of severe inflammatory myocardial damage due to impaired expression of cardiac adiponectin and induced expression of TNF-α (Takahashi et al., 2006). Inbred male C57BL/Ks homozygous *db/db* mice exhibited higher susceptibility to Coxsackie virus B4 infection than that of heterozygous normal (*db*/+) and normal (+/+) genotypic mice (Webb et al., 1976).

### Cyclic-relationship of malnutrition with parasite infections regulates leptin production

Malnutrition in children is directly linked to the enteric parasite infections, which causes damage to intestinal mucosal epithelial cells by inducing inflammation and ulceration, it eventually leading to multiple pathological conditions like anorexia, indigestion, malabsorption, and nutrient loss (Vermeulen et al., 1998; Saldiva et al., 2002), and responsible to mimic malnutrition. Malnutrition is the hall mark of low systemic leptin levels (Sánchez-Margalet et al., 2003) and gut parasite infections may induce this state. A recent study on Bangladesh population suffering from amoebic liver abscess caused by *E. histolytica*, provides a significant correlation between disease severity and low serum leptin levels to malnutrition (Alam et al., 2016). The reduced systemic leptin levels found in *Taenia taeniaformis* infected mice may be the result of increased hunger rate induced by parasites (Krebs and Kacelnik, 1991; Lõhmus and Sundström, 2004). This infection also tended to produce lower body mass (anorexia) in animals, which is a symptom of malnutrition. A positive correlation between leptin levels and body mass was consistent in normal animals but such a relationship was absent in infected animals, unveiling the possible effect of the parasite on leptin biosynthesis and production (Lõhmus et al., 2012). A similar condition was reported on enteric parasitic infections in humans (Yahya et al., 2016). Helminths evolved many strategies to evade host immunity for their endurance (Maizels and Yazdanbakhsh, 2003); a report suggested that the larval stage of *T. taeniaformis* induced PGE_2_, which inhibited IL-12 and skewed the host immunity to Th2-type (Leid and McConnell, 1983). As was discussed earlier in *S. pneumonia* infection, the elevated levels of PGE_2_ inhibited the infiltration of skin Langerhans cells to the lymph nodes (LNs), which is a crucial step in the initiation of immunity (Maizels and Yazdanbakhsh, 2003).

There are few contradictory reports, which suggest that leptin levels increased during parasitic infection in children (Zaralis et al., 2008). For example, higher serum leptin levels were reported in malaria (Pulido-Mendez et al., 2002) and in gastrointestinal nematode infection by. *Heligmosomoides bakeri* (Tu et al., 2007). It is possible that low endogenous leptin levels in children (Howard et al., 1999) might result in short-time increase of leptin levels due to acute inflammation and production of IL-1β, TNF-α, and IL-6 caused by the gut infections (Noah et al., 1994). A significant increase in leptin levels was observed during *E. histolytica, Strongyloides*, and *Giardia* co-infections. These parasites are reported to cause damage to the gut epithelial cells, which results in the activation of mesenteric LNs and then adjacent adipose tissue to secrete the leptin (Desreumaux et al., 1999). Leptin functions as an eosinophil survival factor in humans (Conus et al., 2005), which plays a key role in the host defense system against gut parasitic infection. The level of eosinophilia indicates the relative severity of the disease due to tissue invasion by the parasites (Park and Bochner, 2010). Co-infection of *E. histolytica* and *Strongyloides* positively correlated with increased leptin suggests it promotes pathogen tissue invasion and pathogenicity. This is however, not found in infections with *Giardia, Hymenolepis nana*, and *Oxyuris*, but reappeared with *E. histolytica* and *Giardia* co*-*infection suggesting that *E. histolytica* is a crucial player of tissue invasion (Yahya et al., 2016).

### LEPR signaling and *E. histolytica* infection

Enteric leptin plays a crucial role in the immunity to *E. histolytica* (Guo et al., 2011). Apart from this, it promotes regeneration and inhibits apoptosis in intestinal epithelium (Sukhotnik et al., 2009), stimulates mucin secretion (El Homsi et al., 2007) and maintains intestinal epithelium integrity (Brun et al., 2007). Its secretion into the gastric juice regulates digestion and absorption (Morton et al., 1998). Furthermore, intact leptin signaling plays a key role in offering protection against gut parasite infections e.g., *E. Histolytica* caused amoebiasis. However, a mutation (E233R) or polymorphism in LEPR is likely to enhance the susceptibility for *E. Histolytica* infection by four-folds in children, irrespective of their nutritional status. In mice, leptin-deficiency has shown intermediate susceptibility and leptin-receptor deficiency has shown high susceptibility to this amoeboid infection, and mice ceca developed intensive epithelial denudation. An integral leptin signaling in intestinal epithelial cells via STAT3 and Erk or Akt pathways was found to be protective against these amoebic infections, which is governed by non-adipokine action of leptin (Vedantam and Viswanathan, 2012).

### Leptin induces protective immunity to intracellular parasite infections

Leishmaniasis is a vector-borne intracellular infectious disease caused by the protozoan *Leishmania* sp., For the first time, we have hypothesized the possible role of leptin in human VL (Dayakar et al., 2011). Thereafter, several studies on the relationship between leptin and disease outcome in leishmaniasis, indicated that exogenous recombinant leptin augmented the host protective immunity with the combination of miltefosine in mouse macrophages (J774.1 cell line) during *in vitro L. donovani* infection (Shivahare et al., 2015). We have also—demonstrated that leptin induced the host protective Th1-type cytokine response in human THP-1 macrophages and PBMCs. Leptin was able to maintain the defense against *L. donovani* infection through the classical activation of macrophages by inducing the phosphorylation of Erk1/2 and Akt kinase (Dayakar et al., 2016). In addition, leptin was shown to induce protective immunity in normal C57BL/6 mice; it reduced the parasite load in visceral organs such as spleen, liver, and bone marrow derived macrophages, induced IgG2a, IFN-γ, IL-12, IL-1β, and nitric oxide (NO) production. However, leptin failed to restore protective immunity and reduce parasite load in the spleen of *ob/ob* mice but succeeded to reduce the parasite load in liver (Maurya et al., 2016). In malnourished BALB/c mice, leptin induced Th1 immune response and IgG2a class-switching against *L. donovani* challenge. In this study, the fascinating outcome is the serum leptin levels significantly reduced by the parasite infection irrespective of malnutrition i.e., systemic leptin levels were reduced in well-nourished infected animals also (Dayakar et al., 2017). We assume that perturbations in host lipid profile could disturb the host immunity and promote the parasite growth and diffusion inside the lymphoid tissues (Ghosh et al., 2013). It could be a novel mechanism exhibited by the *Leishmania* parasite to evade the host immune defense.

### Interaction between leptin and *Candida albicans* infection

Leptin receptor-deficient obese (*fa/fa*) rats show impaired host defense against *C. albicans* infection whereas, in stressed rats this infection caused a prompt reduction in systemic leptin levels and increased TNF-α levels (Rodríguez-Galán et al., 2010). However, it has been suggested that the leptin can activate healing the critical illness in stress-related activities (Bornstein et al., 1998). Leptin regulates the production of endogenous cortisol through hypothalamic-pituitary-adrenal (HPA) axis, which induces stress response, and hematopoiesis.

## Scope and challenges of leptin in vaccines formulation

To the best of our knowledge, several vaccines failed to prove their efficacy in preclinical studies, and the lack of appropriate immuno-adjuvant could be one of the potential reasons. With the advancement of scientific knowledge and innovative technology, it becomes easier to identify and develop novel vaccine or adjuvant tools (Kennedy and Poland, 2011; Poland et al., 2011). The goal of adjuvant is to elicit robust vaccine-specific immune response and immune homeostasis. Leptin is one such molecule, which can restore inflammatory response without eliciting adverse side-effects since it is produced endogenously. For example, a long-term leptin replacement therapy in congenital leptin-deficient children restored the Th1/Th2 cytokine balance and proliferation of lymphocytes, neutrophils, and monocytes (Farooqi et al., 2002). The 7 days of leptin treatment in these children had no effect on body weight loss, though it reduced energy intake (Farooqi et al., 2007). In contrast, the intra-cerebroventricular route of leptin (1 μg/day) infusion for 7 days in C57B6/J and *ob/ob* mice substantially reduced the daily intake of food and body weight (Tschöp et al., 2010). The peripheral leptin administration does not affect either food intake or body weight (Bryson, 2000). It acts directly on immune cells. In consistence with this hypothesis, we observed that the subcutaneous route of recombinant leptin (5 μg/day) administration into the malnourished BALB/c mice infected by *L. donovani* did not alter the amount of food intake or weight gain; in fact, it controlled the further loss of body weight. In addition, an increased infiltration of CD8+ T cells and their activity was observed in the spleen tissue in terms of Granzyme-A expression and cytotoxic T lymphocyte Antigen (CTLA)-4 and programmed death protein (PD-1) repression (Dayakar et al., 2017). Collectively, our study suggests that leptin can be a potential adjuvant tool in kala-azar vaccination strategies. It was also shown that the peripheral administration of recombinant leptin markedly reduced food intake and body weight in *ob/ob* and diet-induced obese mice without apparent toxicity and had no effect on *db/db* mice (Campfield et al., 1995; Halaas et al., 1995). But these observations in wild-type lean mice are quite lesser and had not reported on alteration in metabolic parameters (Pelleymounter et al., 1995), suggesting clinical perspectives of leptin treatment in weight related complications. However, exogenous leptin therapy may not be beneficial to the obese individuals (Heymsfield et al., 1999) having hyperleptinemia and leptin resistance i.e., impaired leptin signaling due to LEPR deficiency or transport saturation. But the administration of chemical chaperons such as 4-phenylbutyric acid (4-PBA) and tauroursodeoxycholic acid (TUDCA) has reduced the ER stress in diet-induced obesity and increased the leptin sensitivity by ten-folds beside to weight loss during high-fat diet (Ozcan et al., 2009). The clinical investigation on obese and type-2 diabetic phenotypes is required to confirm the therapeutic potential of TUDCA as it known to regulate the weight-loss and leptin/insulin sensitivity (Ozcan et al., 2006), and interact directly with immune cells. The molecular chaperon heat-shock protein 60 (HSP60) may have potential role in the regulation of obesity related chronic ER stress and its expression is correlated with circulatory leptin (Märker et al., 2012). In *in vitro*, leptin stimulates the expression of HSP60 (Bonior et al., 2006), which exerts pro/anti-inflammatory response by interacting with TLR-2 and TLR-4, unveiling the interaction between leptin and chaperons. However, the food and drug administration (FDA) approval of TUDCA for the treatment of primary biliary cirrhosis (Invernizzi et al., 1999) and its inhibitory potential against influenza A viral replication by inducing ER stress (Hassan et al., 2012) are to be controversial for its application in leptin/leptin signaling impaired pathologies. Hence, there is a great scope for active research to identify a specific pharmacotherapy candidate to improve leptin signaling in obesity. Targeting SOCS-3 expression and PTPs activity using appropriate inhibitors and implying ER stress reducing measures could help in reversing leptin insensitivity in obesity.

Studies have shown that immature DCs primed with leptin were licensed to skew the immune response toward Th1-type by upregulating the production of IL-12p70 upon CD40 stimulation. It was also able to induce the activity of autologous CD8+ T cells in terms of perforin and IFN-γ production (Mattioli et al., 2008). The reinfusion of leptin-pulsed autologous DCs into the patients with cancer may induce anti-tumor specific CD8+ T cell response by infiltrating into the LNs. In view of this, there is a scope to develop vaccines against multiple infections e.g., HIV using leptin as an adjuvant in immune cell-based therapy (Martín-Romero et al., 2000; Mattioli et al., 2005). Leptin resistance or desensitization of signaling in obesity (BMI ≥ 30 kg/m^2^) and over-nutrition impaired antigen-specific IgG response as well as reduced CD69, IFN-γ, and Granzyme-B in CD8+ T cells to influenza vaccination (Sheridan et al., 2012). Similarly, hepatitis-B vaccination failed due to reduced HBsAg specific IgG response (Weber et al., 1985) and tetanus vaccination also failed due to low anti-tetanus IgG and high IL-6 (Eliakim et al., 2006). Overall impaired antibody response may be due to the sharp reduction in B-lymphocytes count, for example in ob/ob mice, a firm reduction in pre-B-cells count for 21% and immature B-cells count for 12% was noticed. However, this was further repaired by exogenous leptin supply, unveiling the role of leptin in B-cells generation and activation (Claycombe et al., 2008).

Despite the lack of investigations on leptin-based therapies, the immunostimulatory potential of leptin cannot be neglected in vaccines development, as an adjuvant (White et al., 2013). Certain investigators tried to explore the adjuvant role of leptin in mucosal vaccination against a gram-positive bacterial pneumonia caused by *R. equi* infection in foals and immunodeficient humans (Cauchard et al., 2011). In this study, mice were vaccinated with LL-VapA (a native *Lactococcus lactis* vector expressing virulence-associated protein-A of *R. equi*) alone or with the combination of LL-leptin (a recombinant *L. lactis* strain for leptin production) through an intra-gastric or intranasal route against *R. equi*. However, only the co-immunization (combination of LL-VapA + LL-leptin) was able to produce protective immunity, when it was ingested through gastric route. On the other hand, LL-VapA alone was able protect against bacterial challenge when it was vaccinated through intranasal route but the co-immunization has stimulated the immune response. Similarly, the LEPR-deficient mice were not protected by prophylactic vaccination in spite of mild gastritis and pathogen specific antibody response during *H. pylori* infection (Wehrens et al., 2008), indicating the importance of leptin and its signaling in the generation host protective immune response. Leptin signaling in mucosal epithelium of vaccinated stomach possibly trigger the infiltration of effector CD4+ T cells, neutrophils and macrophages, and reduce CD4+ Treg cells to *H. pylori* challenge. CD4+ T cells produced leptin stimulates their own proliferation by autocrine mechanism as well as epithelial cells to induce protective immune response and suppress Tregs mediated pathogenic response (Walduck and Becher, 2012). Acute systemic inflammation induced by *S. typhi* vaccination in humans doubled the plasma IL-6 levels but it did not affect the leptin levels within 24 h, indicating that leptin is not a key molecule in early systemic inflammation (Ekström et al., 2015).

**Figure 2:**
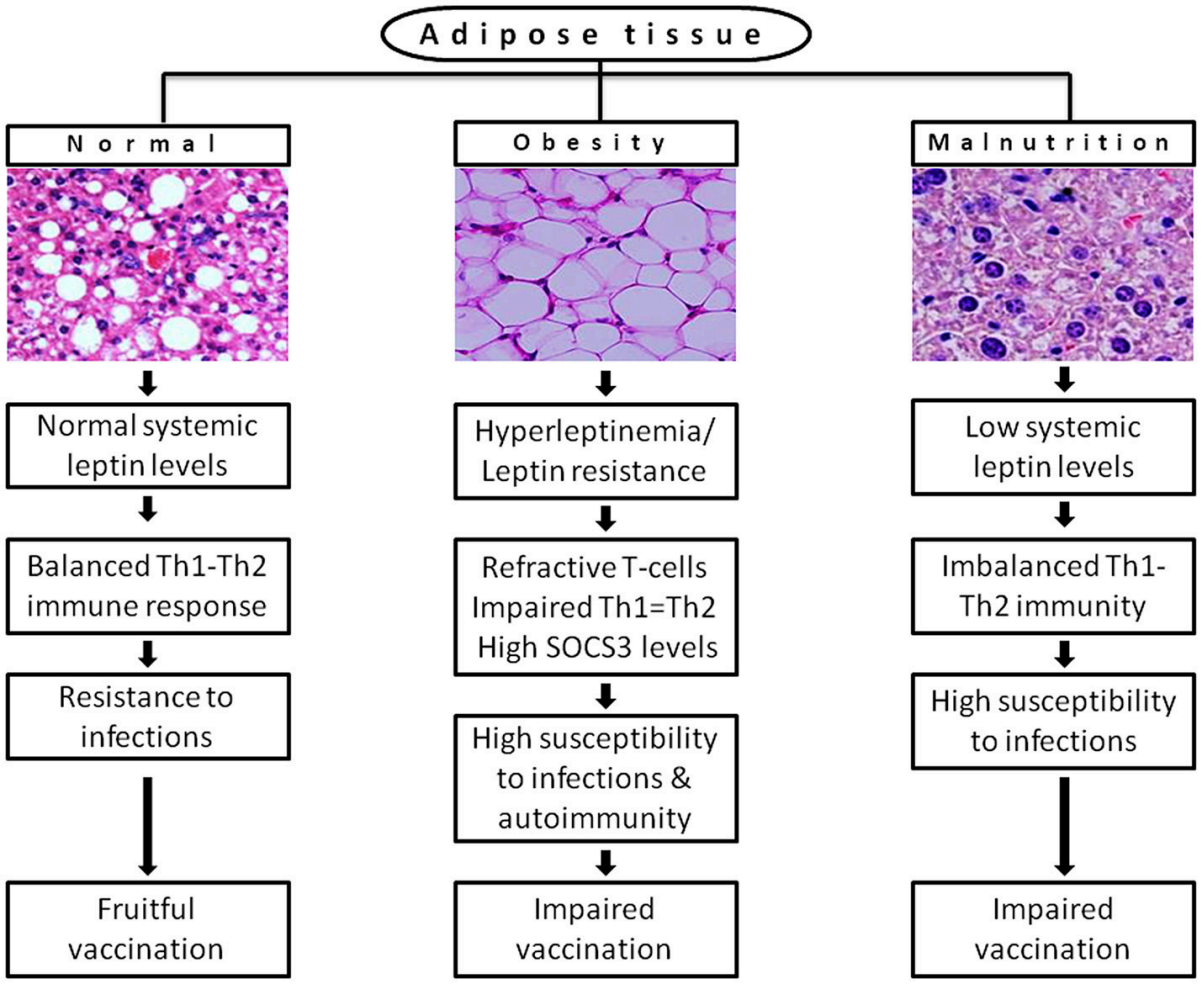
A possible link of differential leptin levels of altered physiological conditions with the rate of susceptibility to infections and vaccination. Adipose tissue of normal individuals produces adequate levels of leptin that offers resistance to multiple infections by maintaining the immune homeostasis. In diet-induced obesity, adipose tissue produces leptin in huge amount (hyperleptinemia) that causes desensitization of target cells for leptin signaling (leptin resistance) results in refractive T-cells response and huge expression of SOCS3, which increases susceptibility to infections and autoimmunity. In malnutrition, reduced mass of adipose tissue produces inadequate systemic leptin that cannot hold the Th1-Th2 balance and increases incidence of multiple infections. Both obesity and malnutrition causes impaired vaccination due to inefficient antibody response and T-cell priming.

In conclusion, the current knowledge suggests a finite role of leptin signaling in immunity, but the data is insufficient at clinical standards. It is based on measuring the concentration of systemic leptin levels, which is not a well-defined marker of actual leptin signaling. In addition, the current eating habits and sedentary lifestyle of developed countries and of urban population in developing countries are becoming real hurdles to control the obesity in children and adults. They are the risk factors for fruitful vaccination attempts to various infections, which emerges in hypo or hyperleptinemia state and in impaired Th1/Th2 balance state shown in Figure 2. However, there is an increasing evidence that leptin is a part of immunopathology of certain physiological conditions. For instance, high systemic leptin in mice associated with T cell mediated hepatotoxicity (Faggioni et al., 2000a) liver fibrosis (Leclercq et al., 2002) autoimmune encephalomyelitis (Matarese et al., 2001; Sanna et al., 2003) intestinal inflammation (Siegmund et al., 2002), coronary heart disease (Yudkin et al., 1999), and type-2 diabetes (Pickup et al., 2000; Matarese et al., 2002). In humans, it associated with pancreatitis (Konturek et al., 2002), sepsis and septic shock (Arnalich et al., 1999) suggests that non-physiological excess levels of leptin are toxic to the host system due to excessive proinflammatory cytokine response and inflammation, which causes tissue damage. The strategies following to preserve the number, proliferation and activity of naturally occurring Foxp3+CD4+CD25+ Tregs may help to counteract the excessive inflammation. As the leptin is a negative regulator of Tregs, the neutralization of leptin with monoclonal antibody in Tregs unlocks their hyporesponsiveness or anergic state by the rapid degradation of cyclin dependent kinase inhibitor p27 (p27^kip1^) and phosphorylation of Erk1/2, and increases IL-2 dependent Foxp3+ Tregs proliferation without altering their suppressive role (De Rosa et al., 2007). This phenomenon may help to protect from autoimmunity but increases susceptibility to intracellular infections. Therefore, the strategic usage of Tregs with the manipulation of leptin signaling may provide a new opportunity to control infectious and autoimmunity disorders. In addition to this, a better understanding the network of underlying mechanisms that connected with the impaired leptin signaling or central vs. peripheral leptin resistance in obesity-related disorders, autoimmunity, and infectious diseases may empower our knowledge toward identifying potential therapeutics to increase the leptin sensitivity as wells as vaccine response to infections. Despite the insulin therapy to improve the cellular uptake of glucose and hyperglycaemia and resulting diabetes in *db/db* individuals, it would be a fascinating discovery if we address a novel strategy to improve leptin signaling in *db/db* cases besides to conventional receptor gene cloning approach in target cells. The lack of functional leptin in *ob/ob* and insufficient leptin in malnutrition or starvation obviously responds to exogenous leptin treatment. However, the route of administration is also critically important. By considering the type of disorder (i.e., metabolic, immunological, endocrine, infectious, and lifestyle) and molecular basis of pathogenicity, leptin can be targeted as a potential therapeutic molecule as well as an adjuvant tool to improve the vaccines efficiency without affecting anti-inflammatory or autoimmune responses.

Our further goal is to find out the correlation between systemic leptin levels and disease severity of VL by using clinical samples of pre/post-treated and disease relapsed patients from endemic regions of India. Though DCs are potential APCs and produce antileishmanial immune response, the studies on DC-based immunotherapy for VL remains low. Antigen-pulsed DCs in conjugation with Antimonial drug was tried against established murine VL. However, the drug conjugation may repeat the complications of toxicity and resistance. The DC-based therapy alone was tried against cutaneous leishmaniasis (CL) but it was proved inefficient to heal the disease, highlighting the importance of an adjuvant in immunotherapy. Therefore, in future prospective, we proposed to investigate the adjuvant potential of recombinant leptin in DC-based immunotherapy for VL.

## Author contributions

SK: Selection of topic, outline of review to be drafted, analysis of leptin interplay with cytokines in infection and immunity, abstract writing and tables information was drafted and edited the overall review to make it to its final shape, and working with references. DA: Literature gathering, preparation of first draft to be corrected by SK, drawing of figures and discussions on view points of scoping leptin and vaccine formulations, and adding proper references to the contexts given in review. CS: Potential discussion and inputs in drafting leptin role of immunity in leishmania.

### Conflict of interest statement

The authors declare that the research was conducted in the absence of any commercial or financial relationships that could be construed as a potential conflict of interest.
